# Constitutive Expression of Insulin Receptor Substrate (IRS)-1 Inhibits Myogenic Differentiation through Nuclear Exclusion of Foxo1 in L6 Myoblasts

**DOI:** 10.1371/journal.pone.0025655

**Published:** 2011-10-03

**Authors:** Fumihiko Hakuno, Yoko Yamauchi, Gen Kaneko, Yosuke Yoneyama, Jun Nakae, Kazuhiro Chida, Tatsuhiko Kadowaki, Keitaro Yamanouchi, Masugi Nishihara, Shin-Ichiro Takahashi

**Affiliations:** 1 Department of Animal Sciences, Graduate School of Agriculture and Life Sciences, The University of Tokyo, Bunkyo-ku, Tokyo, Japan; 2 Department of Bioengineering Sciences, Graduate School of Bioagricultural Sciences, Nagoya University, Nagoya, Aichi, Japan; 3 Frontier Medicine on Metabolic Syndrome, Division of Endocrinology, Metabolism and Nephrology, Department of Internal Medicine, Keio University School of Medicine, Shinjuku-ku, Tokyo, Japan; 4 Department of Veterinary Medical Science, Graduate School of Agriculture and Life Sciences, The University of Tokyo, Bunkyo-ku, Tokyo, Japan; Universita Magna-Graecia di Catanzaro, Italy

## Abstract

Insulin-like growth factors (IGFs) are well known to play essential roles in enhancement of myogenic differentiation. In this report we showed that initial IGF-I signal activation but long-term IGF-1 signal termination are required for myogenic differentiation. L6 myoblast stably transfected with myc-epitope tagged insulin receptor substrate-1, myc-IRS-1 (L6-mIRS1) was unable to differentiate into myotubes, indicating that IRS-1 constitutive expression inhibited myogenesis. To elucidate the molecular mechanisms underlying myogenic inhibition, IGF-I signaling was examined. IGF-I treatment of control L6 cells for 18 h resulted in a marked suppression of IGF-I stimulated IRS-1 association with the p85 PI 3-kinase and suppression of activation of Akt that correlated with a down regulation of IRS-1 protein. L6-mIRS1 cells, in contrast, had sustained high levels of IRS-1 protein following 18 h of IGF-I treatment with persistent p85 PI 3-kinase association with IRS-1, Akt phosphorylation and phosphorylation of the downstream Akt substrate, Foxo1. Consistent with Foxo1 phosphorylation, Foxo1 protein was excluded from the nuclei in L6-mIRS1 cells, whereas Foxo1 was localized in the nuclei in control L6 cells during induction of differentiation. In addition, L6 cells stably expressing a dominant-interfering form of Foxo1, Δ256Foxo1 (L6-Δ256Foxo1) were unable to differentiate into myotubes. Together, these data demonstrate that IGF-I regulation of Foxo1 nuclear localization is essential for the myogenic program in L6 cells but that persistent activation of IGF-1 signaling pathways results in a negative feedback to prevent myogenesis.

## Introduction

Myogenic differentiation is a tightly regulated complex process in which mononucleated myoblasts first proliferate, then withdraw from the cell cycle, differentiate, and fuse to form multinucleated myotubes. Finally, matured myotubes convert into myofibers, which are capable of muscle contraction [1], [2], [3]. This model of differentiation has been extensively investigated using the rat L6 and murine C2C12 myoblast cell lines [4], particularly in the analyses of the myogenic regulatory factors, Myf5, MyoD, myogenin and MRF4 that belong to the basic helix-loop helix (bHLH) transcription factor superfamily [5], [6].

Several extracellular factors are known to modulate myogenic differentiation. Among them, insulin-like growth factors (IGF) -I and -II, potently stimulate myogenic cells to differentiate and are required for the development of skeletal muscle [7], [8], [9]. L6 rat muscle cells are widely used as a model for studying the effects of IGFs on myogenic differentiation because they produce very low amounts of IGF compared with other myogenic cell lines [10]. In myogenic cell lines, IGFs can induce either differentiation or proliferation [7], suggesting that other factors influence myoblast response. Both responses are elicited through binding to the same type 1 IGF tyrosine protein kinase receptor [7]. How a single receptor can elicit two opposite responses is not clear. To address this issue, the IGF-I signal transduction pathways in L6 myogenic cells have been extensively dissected.

IGF-I binding to its specific receptor on plasma membrane activates the IGF-1 receptor intrinsic tyrosine kinase activity [11], [12]. The activated receptor phosphorylates several substrates, including insulin receptor substrates (IRSs) [13], [14]. Phosphotyrosine residues of these substrates are recognized by several SH2 domain containing signaling molecules, including the p85 PI 3-kinase regulatory subunit and Grb2 [13], [15]. These binding interactions lead to the activation of downstream signaling cascades, for example the Ras-MAPK and PI 3-kinase pathways [14], [16]. Active PI 3-kinase generates phosphoinositide 3,4,5 triphosphate (PIP3), resulting in activation of Ser/Thr kinase, Akt [17]. Activated Akt phosphorylates various substrates, including GSK3β, Foxo1 and S6 kinase. Phosphorylation of these substrates is known to play important roles in expression of a variety of IGF-I bioactivities.

It is established that activation of IGF-I signal pathway is required for myogenic differentiation. In addition, there are accumulated reports that impairment of IGF-I signaling through IRSs inhibits myogenic differentiation [18], [19], [20]. However, how IGF-I promotes opposite effects, proliferation and differentiation, and how IGF-I signaling induces myogenic differentiation remained unknown. In this paper, to address these questions, IRS-1 was over expressed in L6 myoblast cells, and myogenic differentiation was studied. Surprisingly, our data demonstrated that prolonged activation of IGF-I signaling did not enhance but inhibited myogenesis.

## Results

### Constitutive expression of IRS-1 inhibits myoblast differentiation

**Figure 1:**
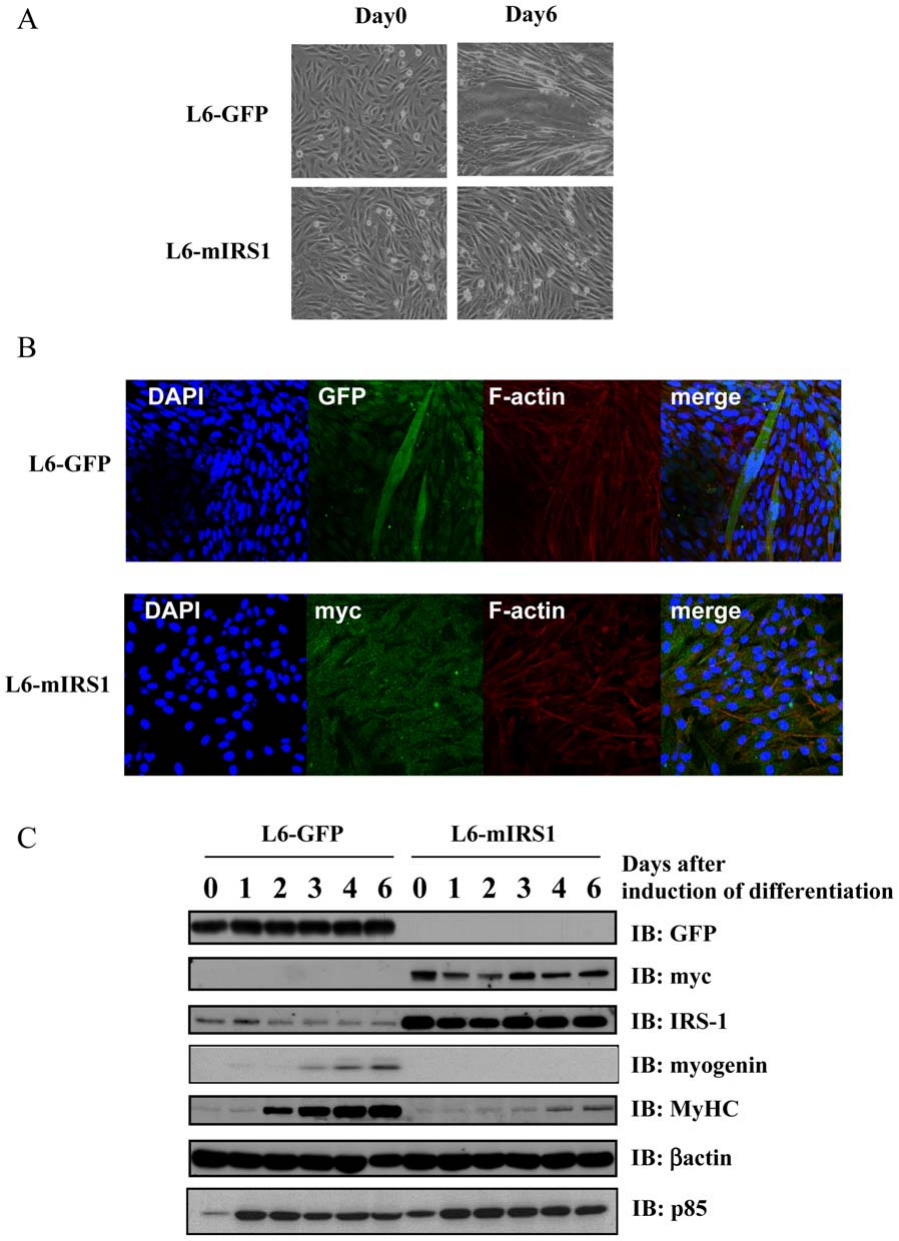
Effects of IRS-1 constitutive expression on myogenic differentiation in L6 myoblasts. A: Differentiation of L6 myoblasts stably expressing GFP (L6-GFP) or myc-IRS1 (L6-mIRS1) was induced by changing medium from 10% FBS-DMEM to 2% FBS-DMEM. At 0 or 6 days after induction of differentiation, cell morphology was shown. B: Differentiation of L6-GFP or L6-mIRS1 cells was induced. Cells were fixed on 6 days after induction of differentiation and stained with DAPI (blue) or phalloidine (red). C: Differentiation of L6-GFP cells or L6-mIRS1 cells was induced. Cells were lysed on the indicated day (0, 1, 2, 3, 4 or 6: days after induction of differentiation). Ten µg of total cell lysates was separated by SDS-PAGE, and subjected to immunoblotting analyses with indicated antibodies (*IB*). These are representative immunoblots independently performed three times.

To examine a role of IRS-1 in L6 differentiation, IRS-1 was over expressed in L6 myoblast cells by retroviral infection. L6 cells stably expressing control GFP (L6-GFP) or myc-tagged IRS-1 (L6-mIRS1) was selected and multiple independent clones were analyzed for expression of GFP or myc-IRS1 by immunoblotting. Three independent lines were analyzed and results shown are representative of these isolates. Expression level of IRS-1 in L6-mIRS1 was 8–10 fold that in L6-GFP (Fig. 1C). At first, ability of these lines to differentiate into myotubes was assessed. L6-GFP or L6-mIRS1 lines were induced to differentiate by exchanging medium containing only 2% FBS. L6-GFP exhibited fusions indicated by multinucleated myotube formation, whereas L6-mIRS1 cells did not fuse with each other and only displayed mononucleated cells (Fig. 1A, B). Immunoblotting analyses indicated that early myogenic marker protein, myogenin, and late myogenic marker protein, myosin heavy chain (MyHC) expression was induced in L6-GFP control cells after differentiation. On the contrary, protein expression of myogenin or MyHC was significantly suppressed in L6-mIRS1 cells (Fig. 1C, S1A). mRNA expression of myogenin or MyHC was also suppressed in L6-mIRS1 cells (Fig. S1B). In addition, on 8, 12 and 15 days after induction of differentiation, expression of myogenin and MyHC were suppressed in L6-mIRS1 cells (Fig. S1C). These indicated that constitutive expression of IRS-1 inhibited myogenesis in L6 cells.

### Constitutive expression of IRS-1 did not enhance IGF-I induced proliferation

**Figure 2:**
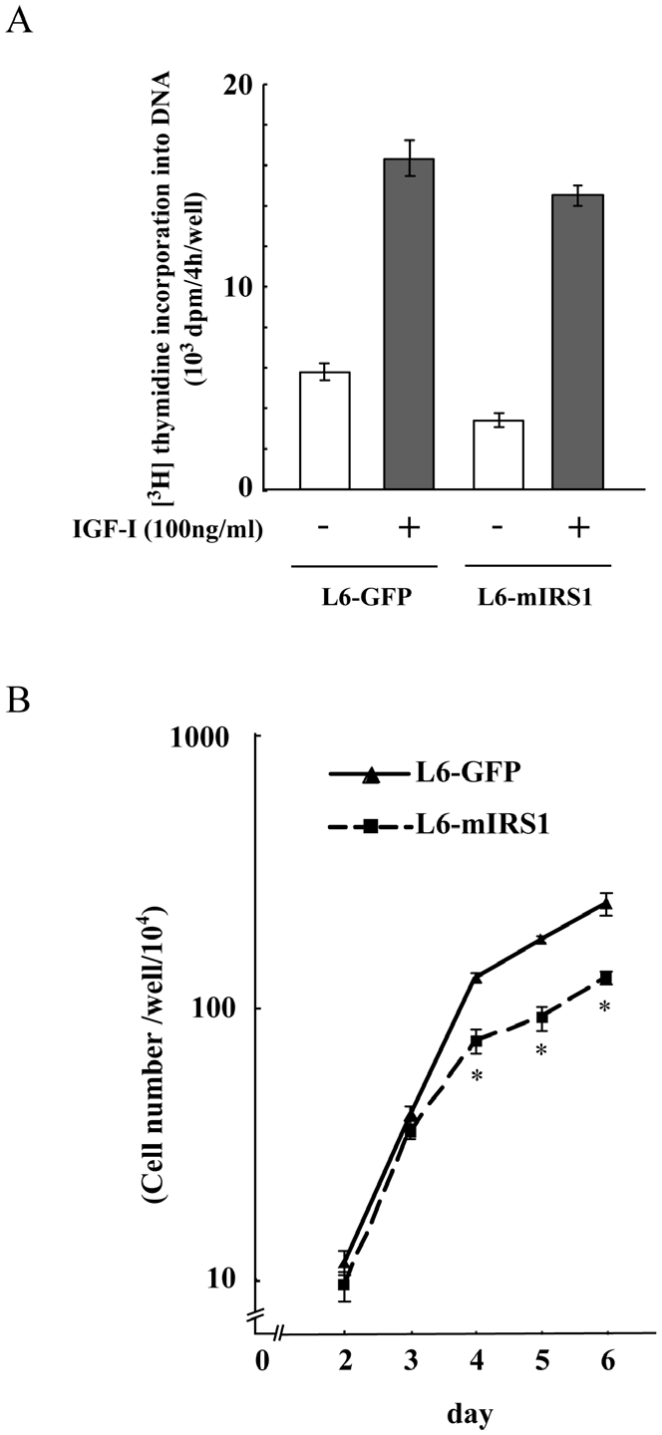
Effects of IRS-1 constitutive expression on cell growth. A: [Methyl-^3^H] thymidine incorporation into DNA was measured during the last 4 h of IGF-I treatment time. The mean ± SEM of three replicate dishes is shown. B: 3×10^3^ cells of L6-GFP control or L6-mIRS1 cells were inoculated in 35 mm dishes. Cells were grown in DMEM containing 10% FBS and cell number was counted in each day. *, difference between L6-GFP control cells and L6-mIRS1 cells is significant with p<0.05.

We then investigated the mechanisms of myogenic inhibition by constitutive expression of IRS-1. Because IGF-I is known to induce cell growth, we had hypothesized that L6-mIRS1 did not withdraw from cell cycle due to the enhancement of IGF-I signaling. At first we measured DNA synthesis in L6-GFP or L6-mIRS1 cells. As shown in Fig. 2A, IGF-I-induced DNA synthesis was not enhanced but in fact appeared somewhat reduced in L6-mIRS1 compared to the L6-GFP cells, although not statistically different. Similarly, as shown in Fig. 2B, cell growth was also not enhanced but again was somewhat reduced in the L6-mIRS1 cells compared to the L6-GFP cells. In addition, L6-mIRS1 cells stop growing at a lower cell density than the L6-GFP cells. Based upon these results, we conclude that the inability of L6-mIRS1 to differentiate is not due to a block of cell cycle withdrawal.

### Constitutive expression of IRS-1 maintained IRS-1 protein level resulting in prolonged Akt activation

**Figure 3:**
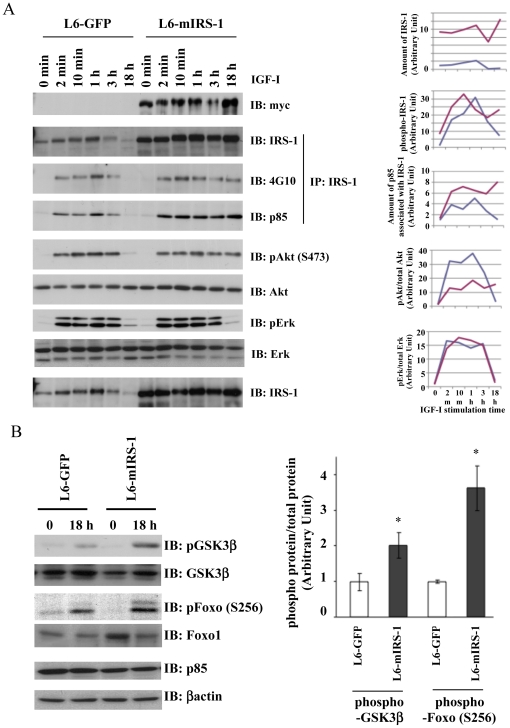
Effects of IRS-1 constitutive expression on IGF-I signal activation in L6 myoblasts. A, B: L6-GFP cells or L6-mIRS1 cells were serum starved for 8 h, followed by stimulation with IGF-I (100 ng/ml) for indicated time (0 min, 2 min, 10 min, 1 hour, 3 hour and 18 hour). Cells were harvested and lysed in RIPA buffer. One hundred µg of cell lysates were immunoprecipitated with anti-IRS-1 antibody (*IP*). Ten µg of total cell lysates or immunoprecipitates were separated with SDS-PAGE and immunoblotted with indicated antibodies (*IB*). Bands were quantified from each blot by NIH Image J software. Protein amount of IRS-1, p85 associated with IRS-1, phosphorylated IRS-1 or phosphorylated proteins over total proteins (pAkt/Akt, pErk/Erk, pGSK3β/GSK3β and pFoxo1/Foxo1) was calculated and the values were shown in the graphs. B: Values are the mean ± SEM of three different experiments and expressed as relative to data in insulin-stimulated L6-GFP cells. *, the difference between L6-GFP cells and L6-mIRS1 is significant with p<0.05.

To examine IGF-I signaling control L6-GFP and L6-mIRS1 cells were serum starved for 8 hours followed by stimulation with 100 ng/ml IGF-I for indicated times (Fig. 3A). Acute IGF-I stimulation (2 min, 10 min, 1 h and 3 h) resulted in IRS-1 tyrosine phosphorylation and association of IRS-1 with p85 PI 3-kinase regulatory subunit. These changes were somewhat enhanced by IRS-1 constitutive over expression. However, neither Akt nor ERK phosphorylation was affected. Acute signal activation with various concentrations of IGF-I was also measured (Fig. S2). With lower concentrations of IGF-I stimulation, enhancement ratio of Akt or Erk phosphorylation by IRS-1 over-expression was also very low, suggesting that IRS-1 high-level expression did not much affect acute downstream signal activation. On the other hand, in L6-mIRS1 cells, IGF-I-induced IRS-1 tyrosine phosphorylation was maintained at 18 h after IGF-I stimulation, whereas in L6-GFP cells IRS-1 tyrosine phosphorylation was suppressed at 18 h. Reflecting this prolonged IRS-1 tyrosine phosphorylation, the association of IRS-1 with p85 PI 3-kinase regulatory subunit was also maintained in L6-mIRS-1 cells. PI 3-kinase activation is well known to induce phosphorylation and activation of Akt kinase. Consistent with the persistent PI 3-kinase activation, Akt phosphorylation (Ser 473) was also sustained at 18 h after IGF-I stimulation in the L6-mIRS-1 cells. To elucidate the molecular mechanism of this prolonged activation of IGF-I signaling, IRS-1 protein level was measured in both cells. As expected, IRS-1 protein level was substantially higher in L6-mIRS1 compared with L6-GFP control cells. In addition, in L6-GFP control cells, IGF-I stimulation led to IRS-1 degradation, and therefore IRS-1 protein levels were significantly reduced following 18 h of IGF-I stimulation. On the other hand, IRS-1 protein levels in the L6-mIRS1 cells were maintained throughout the 18 h IGF-1 treatment time course. The transcriptional factor, Foxo1 is a well-established substrate of activated Akt kinase. GSK3β kinase is also phosphorylated by activated Akt kinase. As shown in Fig. 3B, Foxo1 phosphorylation 18 h after IGF-I stimulation was significantly increased in L6-mIRS1 compared to L6-GFP cells. Similarly, the L6-mIRS1 cells also displayed enhanced GSK3β phosphorylation.

### IRS-1 expression results in nuclear exclusion of Foxo1

**Figure 4:**
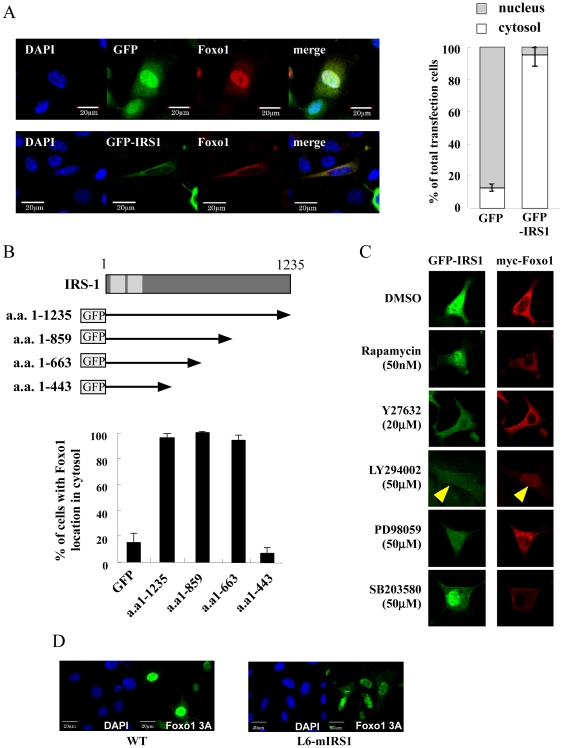
Effects of IRS-1 expression on Foxo1 localization. A: L6 myoblasts were transfected with pGFP or pGFP-IRS1 along with pmyc-FoxoH215R. Eighteen hours after transfection, cells were fixed and immunostained with myc antibody. Foxo1 localization was shown in red. Among the GFP or GFP-IRS1 expressing cells, percentage of cells with myc red signals in the cytosol was calculated and shown in the right graph. Values are the mean ± SEM of at least three different experiments. B: Schematic structure of IRS-1 was shown. Below this, series of IRS-1 deletion constructs fused with GFP were shown. L6 myoblasts were transfected with plasmids expressing series of IRS-1 deletion constructs along with plasmids expressing myc-FoxoH215R. Percentage of Foxo1 cytosolic localization was shown in the graph. Values are the mean ± SEM of at least three different experiments. C: L6 myoblasts were transfected with pGFP-IRS1 along with pmyc-FoxoH215R. One day after transfection, indicated inhibitor was added into the medium. One day after inhibitor addition, cells were fixed and immunostained with anti-myc antibody. GFP-IRS1 localization was shown in green and Foxo1 localization was shown in red. D: L6 cells or L6-mIRS1 cells were transfected with pGFP-Foxo1 3A. Cells were fixed and stained with DAPI. Nuclear staining is shown in blue and Foxo1 3A is shown in green.

Since Akt-dependent Foxo1 phosphorylation results in its nuclear export, we next determined Foxo1 localization. We established an assay system to measure the ratio of cytosolic to nuclear Foxo1 in L6 cells. His residue at 215 in Foxo1 was reported to be required for its transcriptional activation [21]. To exclude the possibility that Foxo1 transcription activity affected Foxo1 localization, we generated a FoxoH215R mutant in which His^215^ was substituted with Arg. L6 cells were transfected with pGFP-IRS1 or pGFP along with pmyc-FoxoH215R. Cells were maintained in 10% FBS medium for 18 h, and immunostained with anti-myc antibody. As shown in the images of Fig. 4A, Foxo1 was localized in the nuclei in GFP-expressing control cells. However, Foxo1 was mainly in the cytosol in the GFP-IRS1 expressing cells (Fig. 4A). These data indicated that GFP-IRS-1 expression excluded Foxo1 from the cells nuclei. We next examined the effect of an IRS-1 deletion series on Foxo1 localization (Fig. 4B). Full length of IRS-1 excluded Foxo1 from nuclei as did IRS-1 containing the amino terminal 859 and 663 amino acids, whereas the 1–443 amino acid construct was unable to affect Foxo1 localization (Fig. 4B). Since sequence between 443–663 contains five p85 binding motifs, these data are consistent with IRS-1 associated PI 3-kinase as necessary for nuclear exclusion of Foxo1. To further examine the specificity for PI 3-kinase, the addition of the PI 3-kinase inhibitor LY294002 during incubation in 10% FBS inhibited the IRS-1 induced nuclear export of Foxo1 (Fig. 4C). In contrast, inhibitors of other signaling pathways, rapamycin (mTOR inhibitor), Y27632 (Rho kinase inhibitor), PD98059 (MEK inhibitor) and SB203580 (p38 inhibitor) during incubation in 10% FBS, had no significant effect on IRS-1 induced Foxo1 nuclear export.

It was reported that Foxo1 3A mutant, in which three Ser/Thr residues phosphorylated by Akt kinase were substituted with Ala, was located in nuclei irrespective of IGF-I signaling [22], [23]. L6 myoblast or L6-mIRS1 was co-transfected with the pGFP-Foxo1 3A mutant. As shown in Fig. 4D, constitutive expression of IRS-1 had no effect on the nuclear localization of the Foxo1 3A mutant. These data demonstrated that IRS-1 over expression results in nuclear exclusion of Foxo1 in a PI 3-kinase-Akt-dependent manner.

### Stable expression of dominant negative form of Foxo1 (Δ256Foxo1) inhibited myogenesis in L6 cells

**Figure 5:**
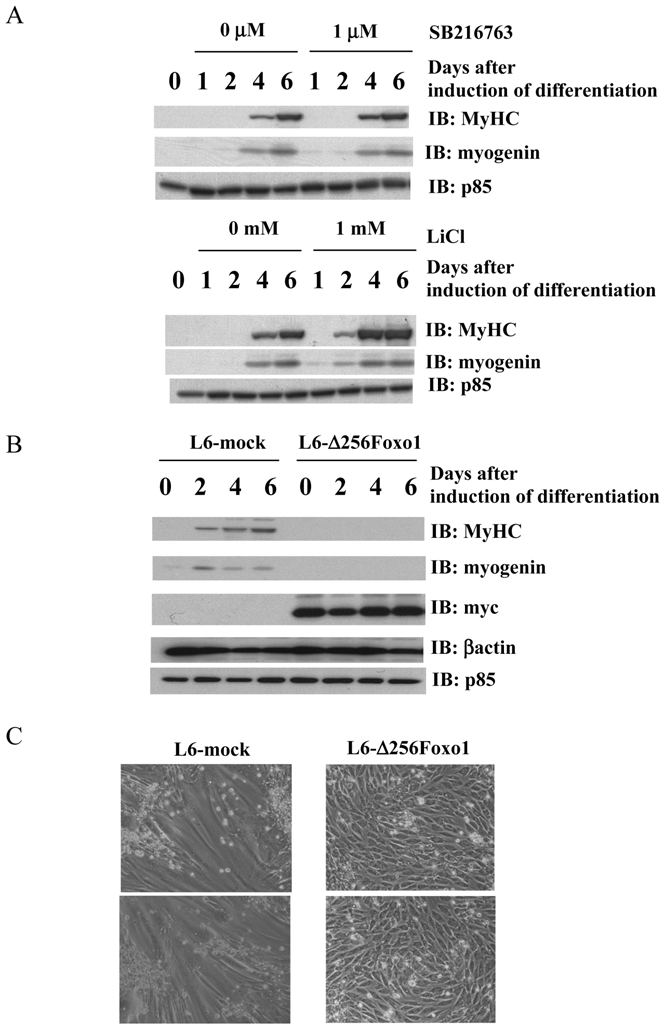
Effects of Foxo1 (Δ256) expression on myogenic differentiation. A: Differentiation of L6 myoblast cells were induced by changing medium from 10% FBS-DMEM to 2% FBS-DMEM. During induction of differentiation, various concentrations of SB216763 (a specific inhibitor to GSK3) or LiCl were added to the medium. Cells were harvested at the indicated day after induction of differentiation. Immunoblotting analyses were carried out using indicated antibodies (*IB*). These are representative immunoblots independently performed three times. B: Differentiation of L6 myoblast cells stably expressing dominant interfering form of Foxo1 (L6-**Δ**256Foxo1) and cells infected with mock retrovirus vector (L6-mock) were induced by exchanging medium containing 2% FBS from 10% FBS. Cells were lysed on the indicated day (0, 1, 2, 4 or 6: days after induction of differentiation). Ten µg of total cell lysates were separated by SDS-PAGE, and subjected to immunoblotting analyses with indicated antibodies (*IB*). These are representative immunoblots independently performed three times. C: At 6 days after induction of differentiation, cell morphology was shown.

Since GSK3β and Foxo1 phosphorylation were enhanced in L6-mIRS-1 myoblast cells, we speculated that persistent phosphorylation of these substrates might account for the inhibition of myogenesis. It is well known that activity of both Foxo1 transcription factor and GSK3β kinase are inhibited by Akt kinase phosphorylation. To examine the roles of these Akt substrates in myogenesis, a specific inhibitor against GSK3 was added into differentiation medium. Addition of specific inhibitor, SB216763 or LiCl did not inhibit but somewhat enhanced myogenic differentiation (Fig. 5A, Fig. S3). These data indicated that prolonged inhibition of GSK3β in L6-mIRS1 was unlikely to account for myogenic inhibition.

Previous studies have reported that a Foxo1 mutant, **Δ**256Foxo1, lacking 256 N-terminus residues including transcriptional activation domain and Akt phosphorylation sites functions as a dominant negative mutant [22]. Stable L6 cell lines expressing **Δ**256Foxo1 (L6-**Δ**256Foxo1) were established and expression of this mutant construct was confirmed (Fig. 5B). Control L6-mock and L6-**Δ**256Foxo1 cells were induced to differentiate by changing medium. As shown in Fig. 5C, expression of L6-**Δ**256Foxo1 was a potent suppressor of L6 cell fusion. Myogenic marker protein expression including myogenin and MyHC were significantly suppressed in the L6-**Δ**256Foxo1 cells (Fig. 5B, Fig. S1A). In addition, mRNA levels of myogenin and MyHC were also suppressed in the L6-**Δ**256Foxo1 cells (Fig. S1B). These data indicated that expression of dominant negative form of Foxo1 inhibited myogenic differentiation. Thus, these data support a model in which Foxo1 exclusion from the nuclei is at least one of the mechanisms responsible for myogenic inhibition by IRS-1 overexpression.

### Endogenous Foxo1 is localized in the nuclei when myogenic differentiation is induced

**Figure 6:**
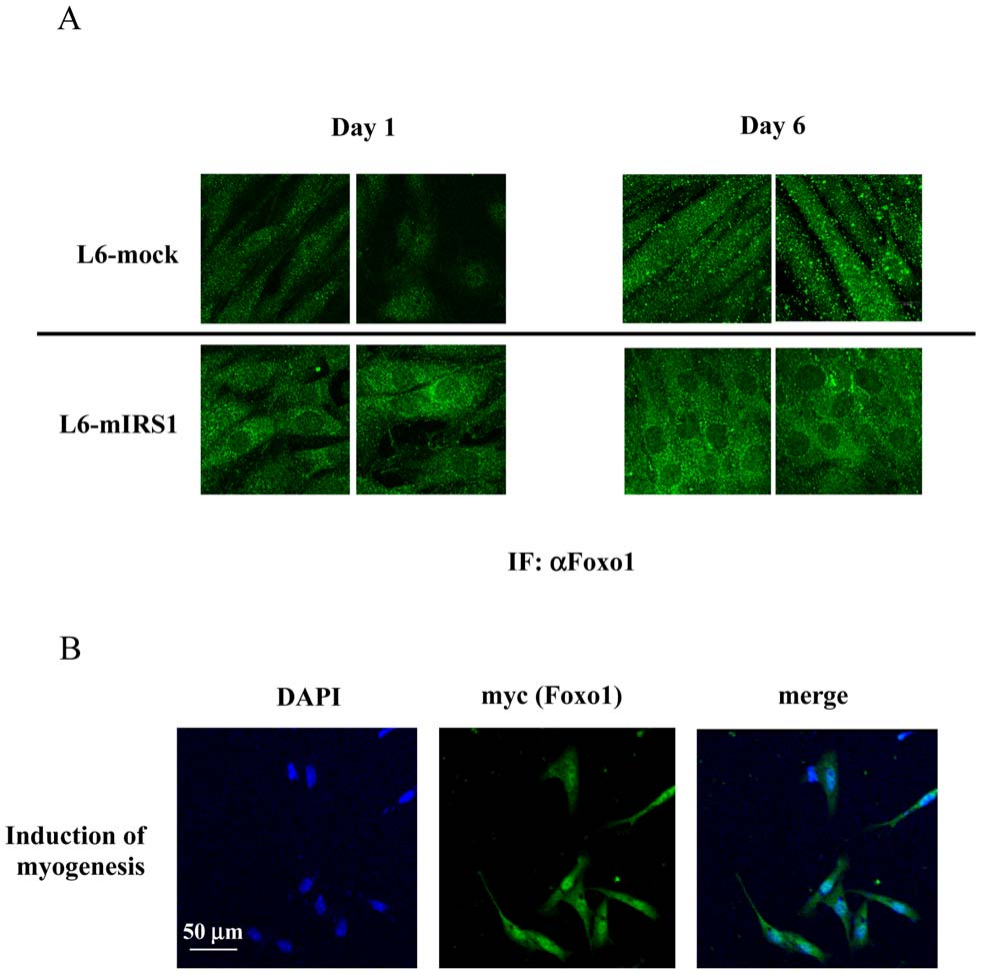
Foxo1 localization in L6 and satellite cells. A: L6-mock or L6-mIRS1 was incubated in DMEM containing 2% FBS for 18 h and 6 days. Cells were immunostained with anti-Foxo1 antibody. Foxo1 localization is shown. B: Satellite cells were separated from the rat soleus muscle and incubated in DMEM containing 20% FBS. Plasmid expressing myc-tagged Foxo1 H215R mutant was transfected into satellite cells. One day after transfection, muscle differentiation was induced by changing medium to 2% FBS. One day after induction of differentiation, cells were fixed, permeabilized and immunostained with anti-myc antibody. DAPI staining was shown in blue. Myc staining (Foxo1) is shown in green.

We have shown that IRS-1 expression resulted in nuclear exclusion of transfected Foxo1, leading to myogenic inhibition. We therefore examined endogenous Foxo1 localization in L6-mIRS1 and L6 control cell during induction of myogenesis. Both cells were incubated with 2% FBS for 18 h or 6 days followed by immunostaining with anti-Foxo1 antibody. As shown in Fig. 6A, Foxo1 was diffusely localized to both nuclei and cytosol in L6 control cells, whereas Foxo1 was localized mainly in the cytosol in L6-mIRS1 stable cells (Fig. 6A). This cytosolic localization was correlated with prolonged enhancement of Akt activation and Foxo1 phosphorylation in L6-mIRS1 cells.

### Foxo1 localization in satellite cells derived from rat muscle

Finally, Foxo1 localization was examined in satellite cells derived from rat muscle. Satellite cells were separated from the soleus muscle as described in ‘Materials and Methods’ and incubated in DMEM containing 20% FBS for 1 day. Isolated satellite cells were transfected with a plasmid encoding myc-tagged Foxo1 H215R mutant. One day after transfection, medium was changed from 20% FBS to 2% FBS in order to induce myogenesis. One day after induction of myogenesis, cells were immunostained with anti-myc antibody. As shown in Fig. 6B, myc-Foxo1 H215R was localized to the nuclei in approximately 80% of the satellite cells in which myogenic differentiation was induced. These data indicated that Foxo1 was localized in the nucleus during the induction of myogenesis.

## Discussion

In this report, we have shown that constitutive expression of IRS-1 inhibited Foxo1 nuclear localization, resulting in inhibition of myogenesis in L6 myoblast cells.

Since IGF-I signaling is moderate in control cells, Foxo1 is mainly localized in the nucleus where it is transcriptionally active as indicated by myogenesis. On the contrary, IGF-I signaling in L6-mIRS1 cells was constitutively elevated and resulted in Foxo1 exclusion from the nucleus where it is transcriptionally active as indicated by myogenesis. As shown in Fig. 5, dominant negative form of Foxo1 expression inhibited myogenesis, indicating that Foxo1 transcriptional activity is required for L6 myogenesis. These data demonstrated that inhibition of Foxo1 transcriptional activity is at least one of the reason why L6-mIRS1 is unable to differentiate. Is exclusion of Foxo1 from nuclei the only reason for myogenic inhibition by IRS-1 constitutive expression? To address this question, a constitutively active mutant of Foxo1, Foxo1 3A was introduced into L6-mIRS1 and ability to differentiate was assessed. Foxo1 3A mutant could not rescue inability of L6-mIRS1 to differentiate into myotubes (data not shown), suggesting that inhibition of Foxo1 transcriptional activity is not the only cause of myogenic inhibition in L6-mIRS1. Prolonged phosphorylation of another Akt substrate rather than Foxo1 could be the target for myogenic defect. As shown in Fig. 3B, phosphorylation of GSK3β, which is one of Akt substrates, was sustained in L6-mIRS1 compared with L6-GFP. So we examined the effect of GSK3β inhibition by adding specific inhibitor, SB216763 or LiCl on myogenic differentiation. SB216763 or LiCl addition did not inhibit myogenesis as detected by expression of MyHC and myogenin (Fig. 5A). Miller *et. al.* reported that addition of LiCl, which is known to inhibit GSK3β, completely inhibited expression of myogenic marker protein and cellular fusion [24]. Although we cannot directly account for these different observations, it is possible that maintenance of another Akt substrate phosphorylation inhibited myogenesis in L6-mIRS1. In addition, as shown in Fig. 2B, L6-mIRS1 cells growth arrested at lower cell density than L6-GFP cells, indicating that an inability to promote withdrawal from the cell cycle is unlikely to account for the inhibition of differentiation.

In this study, Foxo1 activity was shown to be required for myogenic differentiation in L6 cells. However, contrasting findings on Foxo1 role in skeletal muscle differentiation have been reported. In C2C12 cells it was reported that Foxo1 inhibited myogenesis [25], [26]. Kitamura *et al.* reported that a constitutively active form of Foxo1 inhibits differentiation in C2C12 cells [27]. On the other hand, Bois and Grosveld reported that a constitutively nuclear Foxo1 mutant increased myotube formation in primary mouse myoblast cultures [28]. Taken together with our data, these suggested that Foxo1 is required for differentiation at some stages, but at another stage, Foxo1 could inhibit differentiation. To demonstrate this hypothesis, further study is required. Next, what is the role of Foxo1 activity in the myogenesis in L6 myoblast? Foxo1 is known to regulate transcription of various genes including p21 and cyclin D1 that induce cell cycle arrest, MnSOD and catalase for stress response, Gadd45a for DNA repair, and Fas ligand and TRAIL for apoptosis [29]. Expression of p21 increased when myogenic differentiation progressed. p21 could be a candidate for an mRNA which is transcribed in a Foxo1 dependent manner and required for myogenesis. Kitamura *et al.* have reported that Foxo1 ability to regulate skeletal muscle differentiation is mediated through its interaction with Notch. To examine the Notch signaling in L6-mIRS1 cells, we measured mRNA level of Notch target genes, Hes1 or Hes5. As shown in Fig. S4, Hes1 or Hes5 expression was suppressed in L6-mIRS1. These data suggest that Notch target proteins could be good candidates required for myogenesis in L6 myoblast. Further analyses are required for fully evaluate other potential targets downstream of Akt and/or regulated by Foxo1.

Several studies have also reported that IRS-1 protein levels can define insulin/IGF-I signaling intensity [30], [31], [32]. Proinflammatory cytokine, hepatitis C virus infection or retinoic acid treatment was all reported to decrease IRS-1 protein level [33], [34], [35], resulting in suppression of insulin signal activity. These conditions induced the interaction of IRS-1 with ubiquitin ligases including SOCS1, SOCS3, Mdm2 or Cbl-b, leading to degradation of IRS-1 [33], [34], [35], [36], [37]. We also showed, in this report, that IRS-1 protein level was decreased by IGF-I stimulation, leading to suppression of IGF-I signal activation. And this suppression of IGF-I signal resulted in Foxo1 transcriptional activation and enabled myogenic differentiation. In our model, similar mechanism of ubiquitination and proteosomal degradation could be involved in the reduction of IRS-1 protein levels. Hribal *et al.* reported that IRS-1 constitutive expression in the same cellular model did not inhibit differentiation [38]. In their clones, expression of IRS-1 was approximately 2.5–3 fold, whereas our model achieved approximately 10-fold expression. As described above, we hypothesized that exogenous IRS-1 expression level should overcome the IGF-I-induced IRS-1 degradation to show the ability to inhibit myogenesis. This could be the reason why different results from ours were observed in the Hribal reports.

It is well established that IGFs or activation of IGF signal transduction is required for myogenesis. It was demonstrated that IGF-I is required for myogenic differentiation *in vivo* [9]. A specific PI 3-kinase inhibitor, LY294002 inhibited myogenesis in L6 myoblasts [39], [40], [41]. In addition, forced expression of a constitutively active form of p110, PI 3-kinase catalytic subunit, promoted myogenesis in C2BP5 myoblasts [41]. This constitutively active form of Akt, myristylated-Akt also enhanced myogenesis in C2BP5 or C2C12 myoblast [25], [41], [42]. However, we also showed that sustained activation of IGF signaling by constitutive expression of IRS-1 did not enhance but inhibited myogenesis. We speculated that low efficient or late timing expression by transient expression in these reports caused the inability to inhibit myogenesis. As described before, myogenesis can be divided into several processes, including proliferation, growth arrest and cell fusion. Some of these early processes are inhibited by IRS-1/PI 3-kinase/Akt activation, but the other late processes are promoted by IRS-1/PI 3-kinase/Akt activation. Our data prompted us to examine whether IRS-1 protein depletion enhances myogenesis or not in this cell line. We could not assess effects of IRS-1 depletion by siRNA on myogenic differentiation because of defects in cell growth (data not shown).

These data not only recapitulate the necessity of IGF signal activation for myogenic differentiation but also demonstrate that Foxo1, which is inhibited by IGF-I signal, is also required for myogenesis. Together, these data indicate that there must be an initial activation of IGF signaling through the PI 3-kinase/Akt pathway leading to Foxo1 nuclear exclusion. However, subsequently with time the signaling system desensitizes resulting in Foxo1 nuclear localization and activation of transcriptional machinery necessary to drive the myogenic program. We further hypothesize that a subset of Akt substrates needs to be phosphorylated whereas phosphorylation of other Akt substrates needs to be inhibited in order to drive the myogenic program. GSK3β could be the candidate for the former type of Akt substrate, and Foxo1 could be the candidate for latter type. We hypothesize that the activity thresholds of these two types of Akt substrates are different. Alternatively, timing of phosphorylation is different for these two types of Akt substrates. Thus, intensity, timing or quality of IGF-I signal activation should be strictly regulated during induction of muscle differentiation.

In summary, we have shown that constitutive expression of IRS-1 leads to persistent IGF-I signaling, resulting in Foxo1 exclusion from the nuclei, leading to inhibition of myogenesis in the L6 myoblast model cell line.

## Materials and Methods

### Materials

Dulbecco's modified Eagle's medium (DMEM) and fetal bovine serum (FBS) were obtained from Nissui Pharmaceutical CO., LTD. (Ibaraki, Japan). Penicillin was obtained from Banyu Pharmaceutical CO., LTD. (Ibaraki, Japan). Bovine serum albumin (BSA) was obtained from SIGMA (St. Louis, MO, USA). Enhanced chemiluminescence (ECL) reagents were from PerkinElmer Life Sciences (Boston, MA, USA). The other chemicals and reagents, unless otherwise noted, were obtained from Nacalai Tesque, Inc. (Kyoto, Japan).

### Antibodies

Polyclonal anti-IRS-1 antibody was raised in rabbit as described previously [43] and the experiments using rabbits were conducted according to the Guidelines for the Care and Use of Laboratory Animals, Graduate School of Agriculture and Life Sciences, The University of Tokyo (P07-158). Anti-β-actin antibody, anti-myosin heavy chain (MyHC) antibody, anti-GFP antibody and anti-myogenin antibody were obtained from Santa Cruz Biotechnology, Inc. (Santa Cruz, CA, USA). Anti-myc antibody, anti-phosphotyrosine antibody (clone 4G10) and anti-PI 3-kinase p85 subunit antibody were from Millipore (Billerica, MA, USA). Anti-Akt antibody, anti-phospho Akt Ser473 antibody, anti-Foxo1 antibody, anti-phospho Foxo1 Ser256 antibody, anti-GSK3β antibody, anti-phospho GSK3β Ser9 antibody, anti-Erk antibody and anti-phospho Erk antibody were purchased from Cell Signaling Technology, Inc. (Danvers, MA, USA). Horseradish peroxidase (HRP)-conjugated secondary anti-rabbit and anti-mouse IgG antibodies were obtained from GE Healthcare (Pittsburgh, PA, USA). Y27632, Rapamycin, LY294002 and SB203580 were from Merk (Darmstadt, Germany). PD98059 was from Cell Signaling. SB216763 was from SIGMA. Alexa Fluor 488- or 596-conjugated secondary anti-rabbit or anti-mouse IgG antibody was from Invitrogen (Carlsbad, CA, USA).

### Cell culture and treatment

L6 rat skeletal muscle cells (American Type Culture Collection: no. CRL-1458) were obtained from American Type Culture Collection. PLAT-E cells for retrovirus production was a kind gift from Dr. Toshio Kitamura (The Institute of Medical Science, The University of Tokyo, Tokyo, Japan) [44]. L6 myoblast cells and PLAT-E cells were maintained in DMEM containing 10% FBS and antibiotics mixture (50 µg/ml streptomycin, penicillin, and 100 µg/ml kanamycin). To differentiate L6 myoblast cells into myotubes, cells were grown to confluency and the medium was then changed to DMEM containing 2% FBS. Cells were then maintained in DMEM containing 2% FBS for 4–8 days to be differentiated. Satellite cells were obtained from the rat soleus muscle according to Allen *et al* [45]. Isolated satellite cells were incubated in DMEM containing 20% FBS and antibiotics described above. Myogenic differentiation of the satellite cells was induced by changing the media to DMEM containing 2% FBS. These experiments using rats were conducted according to the Guidelines for the Care and Use of Laboratory Animals, Graduate School of Agriculture and Life Sciences, The University of Tokyo (P07–036).

### Transient transfection of L6 myoblasts

The expression plasmids, pGFP-IRS1(1–1235), pGFP-IRS1(1–859), pGFP-IRS1(1–663) and pGFP-IRS1(1–443) were constructed as described before [46]. Foxo1 3A mutant was constructed as described before [23]. A plasmid expressing myc-tagged FoxoH215R, pmycFoxoH215R, was constructed as follow. Site-directed mutagenesis of Foxo1 was carried out using PCR primer, 5′- AG AAT TCA ATT CGC CGC AAT CTG TCC CTT CAC -3′ and 5′- GTG AAG GGA CAG ATT GCG GCG AAT TGA ATT CT -3′. The expression plasmids were transfected into L6 myoblast cells by lipofectamine 2000 (Invitrogen USA) followed by instruction of the kit.

### Isolation of L6 stable transfectant

At first we made the construct of pMX vector containing myc-IRS1 (pMX-mIRS1), GFP (pMX-GFP) or myc-Foxo1**Δ**256 (pMX-m**Δ**256). These vectors were transfected into PLAT-E cells by lipofectamine 2000 by the manufacture protocol. Two days later, conditioned medium was collected and L6 cells were incubated in the conditioned medium containing 5 µg/ml Polybrene. One day later, 200 µg/ml G418 was added to the medium and incubated for additional 7 days. Colony-forming cells were picked up and expression of exogeneous gene product in selected cells was measured by immunoblotting analysis. We established and analyzed at least three lines of each transfectant. In this study we showed representative data obtained from at least 3 lines.

### Preparation of cell lysates and immunoprecipitation with IRS-1

Cells were lysed at 4°C with ice-cold lysis buffer [1% NP40, 50 mM Tris-HCl pH 7.4, 150 mM NaCl, 1 mM EDTA, 1 mM NaF, 10% glycerol, 20 µg/ml phenylmetylsulfonylfluoride (PMSF), 5 µg/ml pepstatin, 10 µg/ml leupeptin, 100 KIU/ml aprotinin, 1 mM Na_3_VO_4_, and 10 mg/ml *p*-nitrophenyl phosphate], or ice cold RIPA buffer [50 mM Tris-HCl (pH 7.4), 15 mM NaCl, 0.1% SDS, 0.5% deoxycholate, 20 µg/ml PMSF, 5 µg/ml pepstatin, 10 µg/ml leupeptin, 100 KIU/ml aprotinin, 1 mM Na_3_VO_4_, and 10 mg/ml *p*-nitrophenyl phosphate]. Insoluble materials were removed by centrifugation at 15,000×*g* for 10 min at 4°C, and supernatant was prepared as a total cell lysate. For immunoprecipitation, 1 mg protein of total cell lysate was incubated with anti-IRS-1 antibody for 2 h at 4°C and the immunocomplexes were precipitated with 20 µl protein A-Sepharose. These precipitates were extensively washed 3 times with ice-cold lysis buffer. These precipitates or total cell lysates were subjected to SDS-polyacrylamide gel electrophoresis (SDS-PAGE) and immunoblotted with indicated antibodies.

### Immunofluorescence analysis

L6 cells were washed once with PBS, and fixed with a solution containing 4% paraformaldehyde in PBS for 10 min. Cells were then permeabilized by incubating in 0.25% Triton ×100 in PBS for 10 min. Cells were then washed with PBS, and incubated with blocking buffer (3% BSA in PBS) for 1 h at room temperature, and primary antibodies (1∶200 for anti-myc, 1∶100 for anti-Foxo1) were added for 1 hour at room temperature. The samples were again washed with PBS, incubated with a secondary antibody conjugated to Alexa Fluor 488 (1∶1000 dilution) or Alexa 596 (1∶1000 dilution) for 40 min, and washed, and the coverslips were mounted using Vectashield for visualization using confocal fluorescence microscope (OLYMPUS, Tokyo, Japan).

### DNA synthesis assay

Quiescent L6-GFP or L6-mIRS1 cells on 48-well plates were serum-starved for 9 h followed by stimulation with or without 100 ng/ml IGF-I for 18 h. [Methyl-^3^H]thymidine (0.3 mCi/well, 1 mCi/ml; GE Healthcare UK Ltd.) was added to each well 4 h before the termination of each experiment. The labeling was stopped by adding 1 M ascorbic acid. The cells were washed twice with ice-cold PBS and twice with ice-cold 10% trichloroacetic acid. Trichloroacetic acid-precipitated materials were solubilized with 250 ml of 0.2 N NaOH and 0.1% sodium dodecyl sulfate (SDS), mixed into 5 ml clear-sol II (Nacalai Tesque), and the radioactivity was measured by a liquid scintillation counter (Aloka, Tokyo, Japan).

### Analysis of mRNA expression

At 0, 1 and 4 days after differentiation, total cellular RNA was isolated by the TRizol reagent according to the manufacturer's protocol (Invitrogen, Carlsbad, CA). First-strand cDNA was synthesized from 2 µg total RNA with oligo-dT primers using the SuperScript II RT-PCR kit (Invitrogen). To determine expression of myogenin, MyHC, Hes1 and Hes5, first strand cDNA was subjected to PCR. Specific primers, Hes1: 5′-CGGCCAATTTGCTTTCCTCATCC-3′ and 5′-TCAGAAGAGAGAGGTGGGCTAG-3′ Hes5: 5′- AGAAGATGCGTCGGGACCGCAT-3′ and 5′- GGAAGTGGTAAAGCAGCTTCATC-3′ myogenin: 5′-CAAGAAAGTGAATGAGGCCTT -3′ and 5′- TCTGGGAAGGTGACAGACATA-3′ MyHC: 5′-AGGGCGGCAAGAAGCAGATC -3′ 5′-TTGTTGACCTGGGACTCGGC-3′ were used for PCR. GAPDH gene was used as the internal control: 5′- AAGCGCGTCCTGGCATTGTCT -3′ and 5′- CCGCAGGGGCAGCAGTGGT -3′.

### Statistical analysis

Statistical analyses of data were performed using StatView software (Abacus Concepts, Inc., Berkeley, CA). Results are expressed as means ± SEM. For comparisons, the data were analyzed by student's t-test. Differences were considered to be statistically significant at *P*<0.05.

## Supporting Information

Figure S1 **Effects of IRS-1 or Foxo1 (Δ256) expression on myogenic differentiation.** A: Differentiation of L6-GFP, L6-**Δ**256Foxo1 or L6-mIRS1 was induced. Cells were lysed in RIPA buffer on 0, 1 or 6 days after induction of differentiation. Ten µg of total cell lysates were separated by SDS-PAGE, and subjected to immunoblotting analyses with anti-myogenin or anti-MyHC antibody. Bands were quantified from each blot by NIH Image J software and quantified data were shown in the graphs. Values are the mean ± SEM of three different experiments and expressed as relative to data from Day1 or Day6 in L6-GFP cells. *, the difference between L6-GFP cells and L6-mIRS1 or L6-**Δ**256Foxo1 is significant with p<0.05. B: myogenin and MyHC mRNA expression were measured by semiquantitative RT-PCR in L6-GFP, L6-**Δ**256Foxo1 or L6-mIRS1 cells. GAPDH expression was used as a control. C: Differentiation of L6-GFP cells or L6-mIRS1 cells was induced. Cells were lysed on the indicated day (4, 6, 8, 12 or 15: days after induction of differentiation). Ten µg of total cell lysates was separated by SDS-PAGE, and subjected to immunoblotting analyses with indicated antibodies (*IB*).
(TIF)

Figure S2 **Effects of IRS-1 constitutive expression on IGF-I acute signal activation in L6 myoblasts.** L6-GFP cells or L6-mIRS1 cells were serum starved for 8 h, followed by stimulation with indicated concentrations of IGF-I (0, 0.1, 1, 10 and 100 ng/ml) for 5 min. Cells were harvested and lysed by lysis buffer. One hundred µg of cell lysates were immunoprecipitated with anti-IRS-1 antibody (*IP*). Ten µg of total cell lysates or immunoprecipitates were separated with SDS-PAGE and immunoblotted with indicated antibodies (*IB*).
(TIF)

Figure S3 **Effects of SB216763 or LiCl on myogenic differentiation.** A, B: Differentiation of L6 myoblast cells were induced by changing medium from 10% FBS-DMEM to 2% FBS-DMEM. During induction of differentiation, SB216763 or LiCl were added to the medium. Cells were harvested at 0, 1 or 6 days after induction of differentiation. Immunoblotting analyses were carried out with anti-myogenin or anti-MyHC antibody. Bands were quantified from each blot by NIH Image J software and quantified data were shown in the graphs. Values are the mean ± SEM of three different experiments and expressed as relative to data from Day1 in L6 control cells. C: myogenin and MyHC mRNA expression were measured by semiquantitative RT-PCR. GAPDH expression was used as a control.
(TIF)

Figure S4 **Effects of IRS1 or Foxo1 (Δ256) expression on Notch signaling.** Before induction of differentiation, total RNA was extracted from L6-GFP, L6-**Δ**256Foxo1 or L6-mIRS1. Hes1 of Hes5 mRNA expression were measured by semiquantitative RT-PCR. GAPDH expression was used as a control.
(TIF)
